# Antibodies against *Escherichia coli* O24 and O56 O-Specific Polysaccharides Recognize Epitopes in Human Glandular Epithelium and Nervous Tissue

**DOI:** 10.1371/journal.pone.0129492

**Published:** 2015-06-18

**Authors:** Agnieszka Korzeniowska-Kowal, Agata Kochman, Elżbieta Gamian, Anna Lis-Nawara, Tomasz Lipiński, Ewa Seweryn, Piotr Ziółkowski, Andrzej Gamian

**Affiliations:** 1 Ludwik Hirszfeld Institute of Immunology and Experimental Therapy, Polish Academy of Sciences, Weigla 12, 53–114, Wrocław, Poland; 2 Department of Pathology, Wrocław Medical University, Marcinkowskiego 1, 50–368, Wrocław, Poland; 3 Department of Medical Biochemistry, Wrocław Medical University, Chałubińskiego 10, 50–368, Wrocław, Poland; 4 Wroclaw Research Centre EIT+, Stabłowicka 147, 54–066, Wrocław, Poland; University of Helsinki, FINLAND

## Abstract

Lipopolysaccharide (LPS), the major component of the outer membrane of Gram-negative bacteria, contains the O-polysaccharide, which is important to classify bacteria into different O-serological types within species. The O-polysaccharides of serotypes O24 and O56 of *E*. *coli* contain sialic acid in their structures, already established in our previous studies. Here, we report the isolation of specific antibodies with affinity chromatography using immobilized lipopolysaccharides. Next, we evaluated the reactivity of anti-O24 and anti-O56 antibody on human tissues histologically. The study was conducted under the assumption that the sialic acid based molecular identity of bacterial and tissue structures provides not only an understanding of the mimicry-based bacterial pathogenicity. Cross-reacting antibodies could be used to recognize specific human tissues depending on their histogenesis and differentiation, which might be useful for diagnostic purposes. The results indicate that various human tissues are recognized by anti-O24 and anti-O56 antibodies. Interestingly, only a single specific reactivity could be found in the anti-O56 antibody preparation. Several tissues studied were not reactive with either antibody, thus proving that the presence of cross-reactive antigens was tissue specific. In general, O56 antibody performed better than O24 in staining epithelial and nervous tissues. Positive staining was observed for both normal (ganglia) and tumor tissue (ganglioneuroma). Epithelial tissue showed positive staining, but an epitope recognized by O56 antibody should be considered as a marker of glandular epithelium. The reason is that malignant glandular tumor and its metastasis are stained, and also epithelium of renal tubules and glandular structures of the thyroid gland are stained. Stratified epithelium such as that of skin is definitely not stained. Therefore, the most relevant observation is that the epitope recognized by anti-O56 antibodies is a new marker specific for glandular epithelium and nervous tissue. Further studies should be performed to determine the structure of the tissue epitope recognized.

## Introduction

Lipopolysaccharide (LPS) is an endotoxic molecule localized on the cell surface of Gram-negative bacteria. It consists of the toxic component lipid A, the core region and the O-antigenic polysaccharide, which is specific for each serotype [1]. The serotypes O24 and O56 of *Escherichia coli* are characterized by the presence of sialic acid in their lipopolysaccharides. The presence of sialic acid in LPS contributes to the pathogenicity of bacteria by molecular mimicry of bacterial surface molecules to structures present on the surface of host tissues, a mechanism of sharing of a common epitope with host structures [2]. Molecular mimicry is an important microbial strategy to evade control by the host immune system. The O-specific polysaccharides of *E*. *coli* O24 and O56 lipopolysaccharides share a similar sugar sequence motif with the common structure →7)-α-NeuNAc-(2→3)-β-D-Glc(1→. Sialic acid is glycosylated with β-D-Glc*p*NAc in O56 and with β-D-Gal*p*NAc in O24 polysaccharide [3]. A well-known example of molecular mimicry based on sialic acid is similarity of bacterial colominic acid to tissue polysialo-glycoconjugates [4] or *Citrobacter* O37 O-specific polysaccharide cross reacting with human erythrocyte band 3 glycoprotein [5]. Particularly important is expression of sialic acid on the tumor cell surface, implicating a functional contribution to the tumor phenotype. Transformation and metastatic progression are accompanied by changes in the quantity, linkage and types of sialic acids on the tumor cell surface [6]. Therefore, it was interesting to examine reactivity of our antibodies against *Escherichia coli* O24 and O56 O-specific polysaccharides with human tissues. Apart from the understanding of molecular mimicry between bacterial and tissue antigens, also a practical application of the studied antibody is important. Cross-reacting antibodies of this type can be a valuable tool for specific recognition of tissue structures, their differentiation and identification for diagnostic purposes. Our studies indicate that several human tissues are recognized by anti-O24 and anti-O56 antibody, although other distinct epitopes were specifically recognized with both anti-O24 and anti-O56 antibodies. Moreover, only a single specific reactivity could be found with anti-O56 antibody. Several tissues studied were not reactive with either antibody, indicating that cross-reactive antigens are distributed in a tissue-specific manner.

## Materials and Methods

### Bacterial strains and culture conditions

Bacterial strains were obtained from the stock of the Polish Collection of Microorganisms (PCM) at the Institute of Immunology and Experimental Therapy, Wrocław. *Escherichia coli* O24 (PCM 195) and O56 (PCM 2372) were the same as used previously [3]. Bacteria were cultivated in Davis broth medium supplemented with casein hydrolysate and yeast extract (Difco), with aeration at 37°C. After 24 h cells were harvested and freeze dried. Lipopolysaccharides were prepared by phenol-water extraction and purified by ultracentrifugation [7].

### Isolation and analysis of LPS using a method with proteinase K

The original procedure [8] was modified slightly as follows. Bacterial dispersion in phosphate buffered saline (PBS) was adjusted to a homogeneous concentration with optical density A_600_ = 0.3, and then 1.5 ml of this material was centrifuged (13 000g, 4°C, 15 min). Pelleted cells were resuspended with 200 μl of 10 mM buffer Tris-HCl, containing EDTA, glycerol and SDS, boiled for 10 min, and treated with proteinase K at 60°C for 2 h. After removal of the precipitated material, the solution was subjected to polyacrylamide gel electrophoresis in the presence of SDS, with subsequent immunoblotting.

### Preparation of sera

Rabbits were immunized with lyophilized bacteria suspended in PBS, first subcutaneously with a dose of 100 μg dry cells/ml in PBS and then intravenously twice a week with increasing amounts of the bacteria (100 to 6400 μg/ml PBS). One week after the last injection the rabbits were bled and the separated sera were decomplemented by heating (56°C, 30 min) and stored at -20°C [9]. The animal studies were conducted in strict accordance with the ethical guidelines established by the National Ethics Committee and approved by the First Local Ethics Commission at the Institute of Immunology and Experimental Therapy, Polish Academy of Sciences (LKE 53/2009).

### Affinity chromatography purification of antibody using immobilized lipopolysaccharide

Lipopolysaccharide (50 mg) was dissolved in 2% SDS with EDTA and precipitated with ethanol followed by three washes with ethanol. The sediments were dissolved in water, passed through Dowex 50x8 (H+ form) and lyophilized. Dry powders were dissolved in dry DMSO and applied to a silica C18 cartridge equilibrated with DMSO. After washing with DMSO, the solvent was exchanged to a buffer and columns used for affinity purifications. Lipopolysaccharide affinity columns preparation will be described in detail elsewhere (Lipiński et al., manuscript in preparation). Antibodies were isolated from rabbit anti-*E*. *coli* O24 and O56 sera (20 ml) diluted with PBS (1:2, v/v) after salting out with ammonium sulfate (9.6 g of (NH_4_)_2_SO_4_), 4°C, 1 h, centrifugation for 30 min. 3000 x g, 25°C). Pelleted antibodies were dissolved in 5 ml PBS and dialyzed to PBS at 4°C. Antibodies were bound to the LPS immobilized on the affinity column, washed with PBS to remove unbound protein and then eluted with 3 M KSCN in PBS, dialyzed to PBS and stored in 50% glycerol at -20°C.

### SDS-PAGE and immunoblotting

SDS-PAGE was done by the method of Laemmli [10] in a 15% acrylamide gel, as described previously [3]. Briefly, LPS suspension (1 mg/ml) in sample buffer or sample of proteinase K treated bacterial extract was boiled for 5 min and 2 μl samples were applied to the gel. After electrophoresis gels were stained with silver according to Tsai and Frash [11,12]. For immunoblotting, after SDS-PAGE, the gel was blotted electrophoretically to nitrocellulose membrane (Schleicher-Schuell, 0.45 μm) as described [13]. The membranes were incubated overnight at 36°C in rabbit antiserum, diluted 1:200 with 1% (w/v) gelatin, washed with Tris-buffered saline (TBS, 20 mM Tris-HCL, 50 mM NaCl, 0.05% Tween-20, pH 7.5) and then incubated with goat anti-rabbit IgG conjugated with horseradish peroxidase diluted 1:5000 in TBS with 1% (w/v) gelatin, for 1 h at 36°C. The immunoblot was stained with 4-chloro-1-naphthol in the presence of H_2_O_2_.

### Immunohistochemical staining

The human tissue sections were obtained from the tissue bank of Department of Pathology at Wroclaw Medical University after the approval from the Bioethics Committee of the Medical University in Wroclaw (ST-727). Formalin (4%) fixed, paraffin embedded (FFPE) human tissue sections were cut from blocks into 4 μm slices and deparaffinized. The immunoperoxidase technique was performed with the ABC DAKO kit: endogenous peroxidase blocking with blocking reagent; distilled water at room temperature (15 min); citric acid buffer pH 6.0 (2 x 8 min, first time in microwave Daewoo at 350 Watt and at room temperature); TBS (0.05 mmol), pH 7.6 with swine serum 1:50 (0.5 h at room temperature); distilled water; investigated antibody (150 μl/slide, 40°C, overnight); TBS; LSAB reagent (30 min); 3,3’-diaminobenzidine tetrahydrochloride (DAB) (5 min). The slides were counterstained with hematoxylin and mounted under coverslips with resin. Negative controls were carried out with TBS instead of the first antibody. Another control for the staining protocol was performed by blocking primary antibodies with the correlating purified LPS antigen in ratio 1.5/1 (w/w). To test whether reacting antigen has a carbohydrate nature, the tissue specimen was subjected to periodate oxidation before labeling with antibodies, according to the procedure previously described [14].

## Results and Discussion

**Fig 1:**
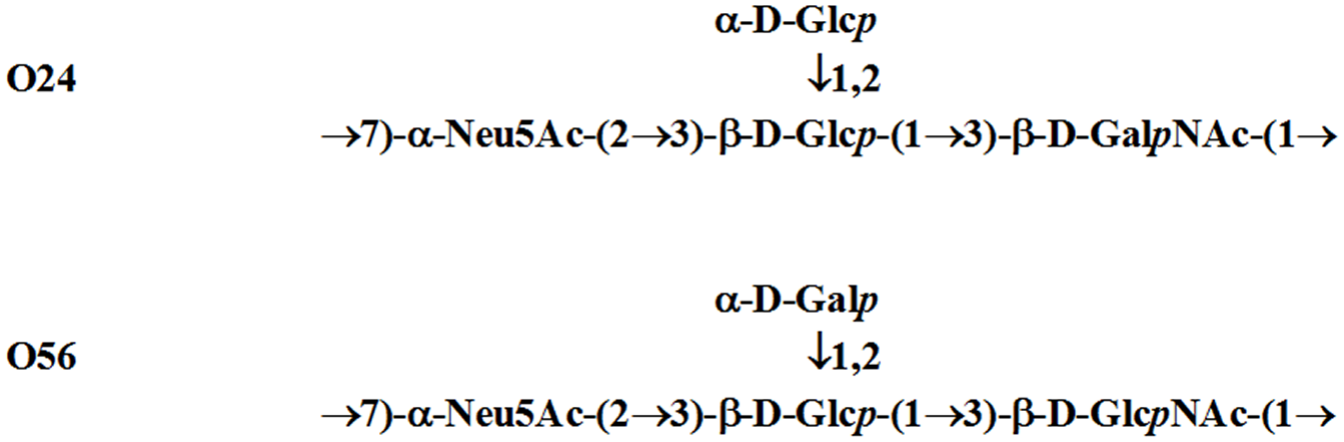
Structures of the O-specific polysaccharides from *E*. *coli* O24 and O56.

**Fig 2:**
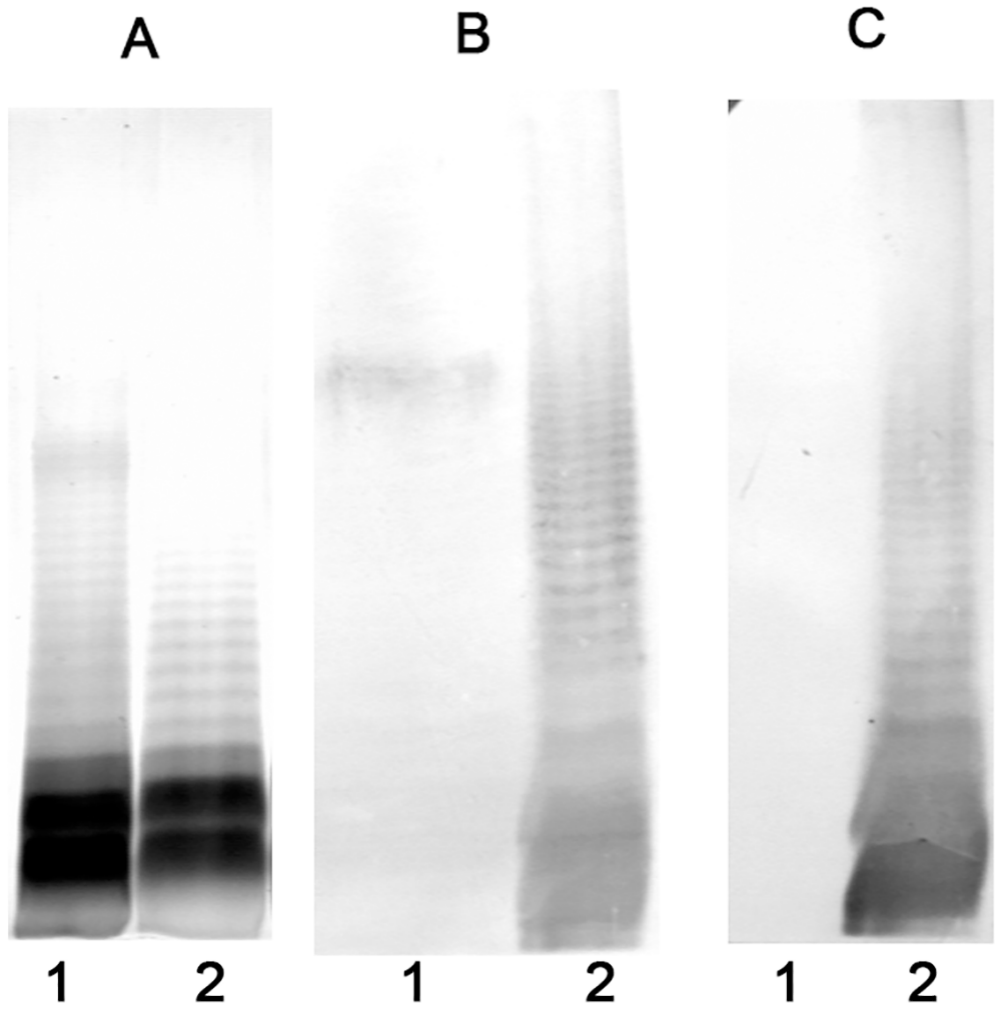
SDS-PAGE analysis of lipopolysaccharides from *E*. *coli* O24 (1) and O56 (2) serotypes (A) and their immunoblotting with affinity purified rabbit antibodies anti-*E*. *coli* O24 (B) and anti-*E*. *coli* O56 (C).

Bacterial cells of *E*. *coli* serotypes O24 and O56 were grown in a liquid medium for the preparation of lipopolysaccharides. Isolation of LPS was performed using a standard hot phenol-water protocol. The method with proteinase K was used for SDS-PAGE analysis. The structures of the sialic acid-containing polysaccharides have been established previously [3] and are shown in Fig 1. Purified LPS was immobilized on a solid phase for the affinity purification of LPS specific antibody. Rabbit polyclonal serum was obtained after immunization with whole bacterial cells. Before affinity purification, the immunoglobulin fraction was obtained by ammonium sulfate precipitation from whole serum. Antibodies bound to immobilized LPS were eluted with KNCS. Fig 2 shows results of SDS-PAGE and immunoblotting analysis of the purified antibody. Clear reaction of antibody with LPS transferred to a membrane is visible. It is noteworthy that anti-*E*. *coli* O24 antibodies showed cross-reactivity with LPS O56, while anti-*E*. *coli* O56 antibodies recognized only homologous LPS. Anti-*E*. *coli* O56 antibodies recognize long chain LPS molecules present in low quantities which were difficult to visualize with silver staining. Interestingly, despite the close structural similarity between both O-antigens and the polyclonal character of serum, anti-O56 antibodies seem to be very specific to an epitope present exclusively on O56 O polysaccharide. The results confirm our previous studies on sialic acid containing lipopolysaccharides isolated from: *Salmonella* toucra O48, *Citrobacter freundii* O37, *Hafnia alvei* PCM 2386, *Escherichia coli* O24 and *Escherichia coli* O104, where we observed cross-reactivity only for anti-O24 serum and O56 LPS [9].

**Fig 3:**
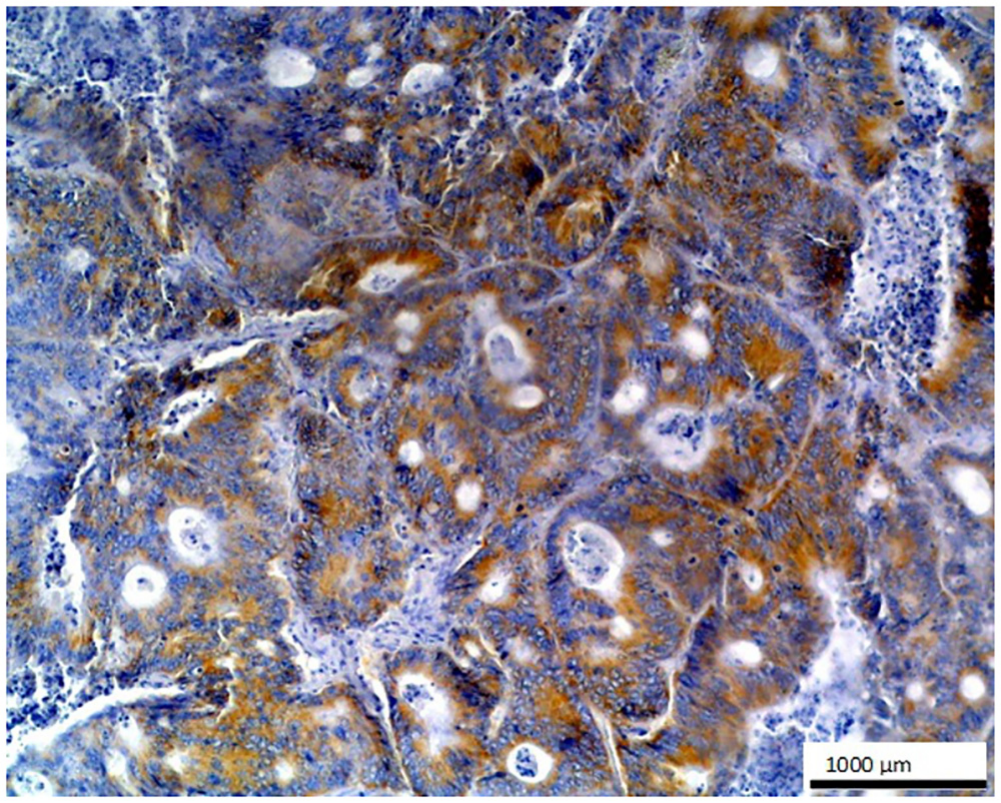
Metastatic colon adenocarcinoma in liver. Strong positive staining is seen in the glandular cells of this metastatic tumor with O56 antibody; LSAB, hematoxylin counterstained, magnification 100x.

**Fig 4:**
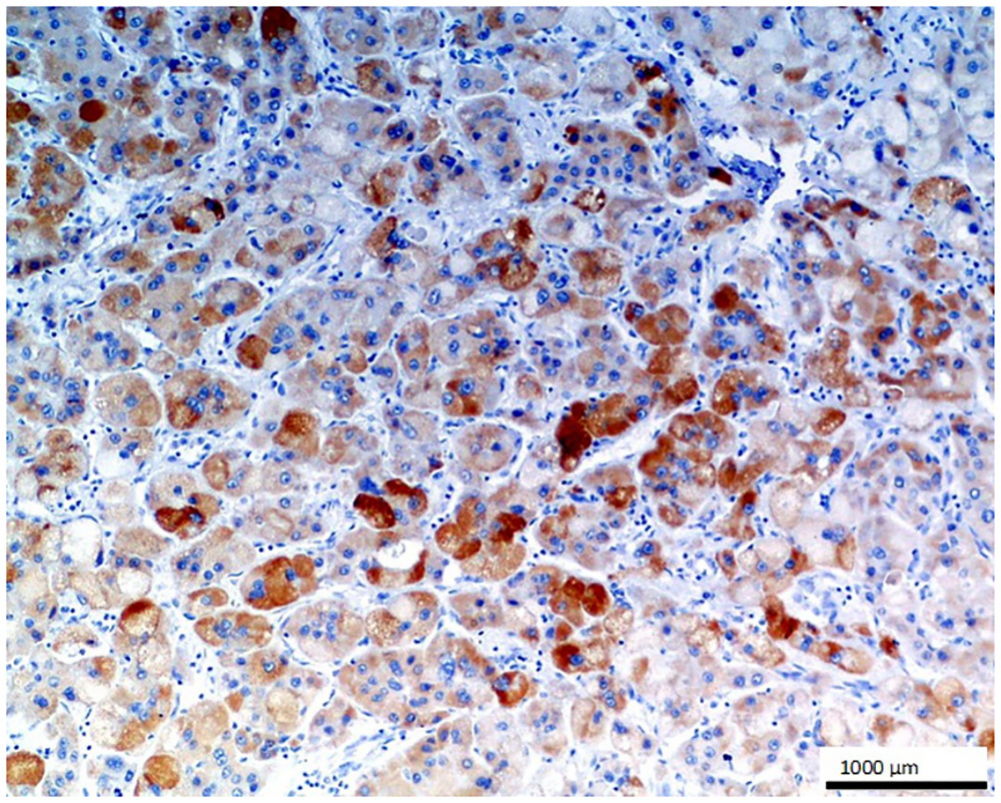
Hepatocellular carcinoma (HCC). The cells of HCC reveal a strong diffuse reaction against O56 antibody; LSAB, hematoxylin counterstained, magn. 100x.

**Fig 5:**
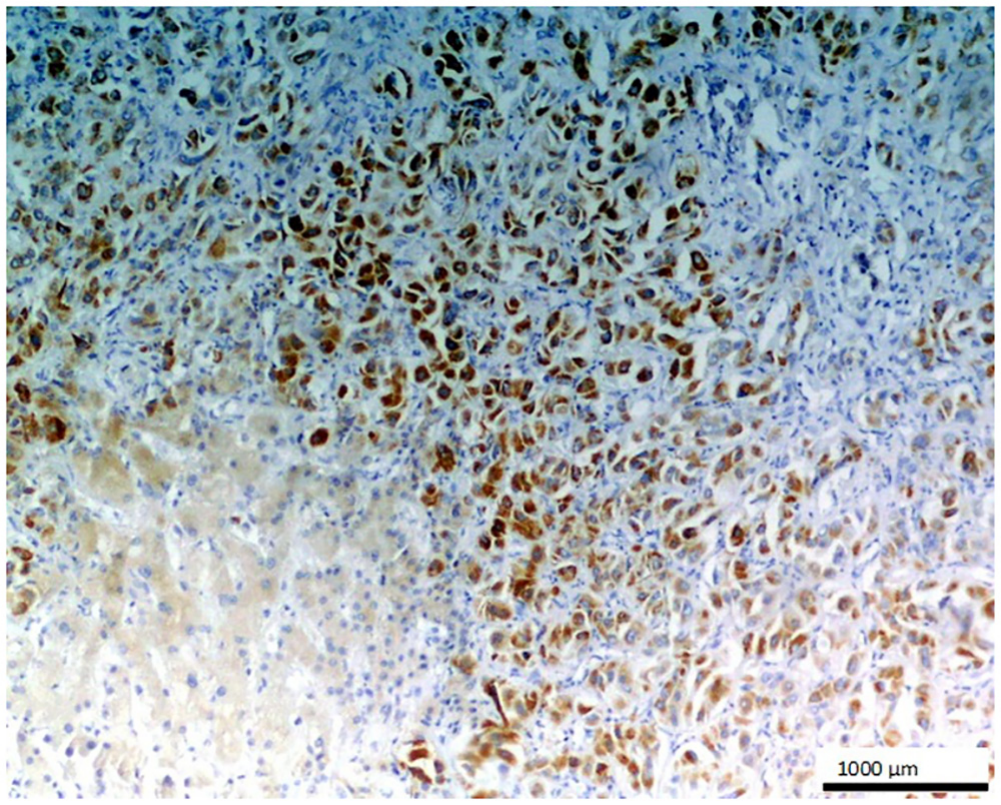
Cholangiocellular carcinoma (CCC). The cells of CCC reveal a very strong diffuse reaction against O56 antibody; LSAB, hematoxylin counterstained, magn. 100x.

**Fig 6:**
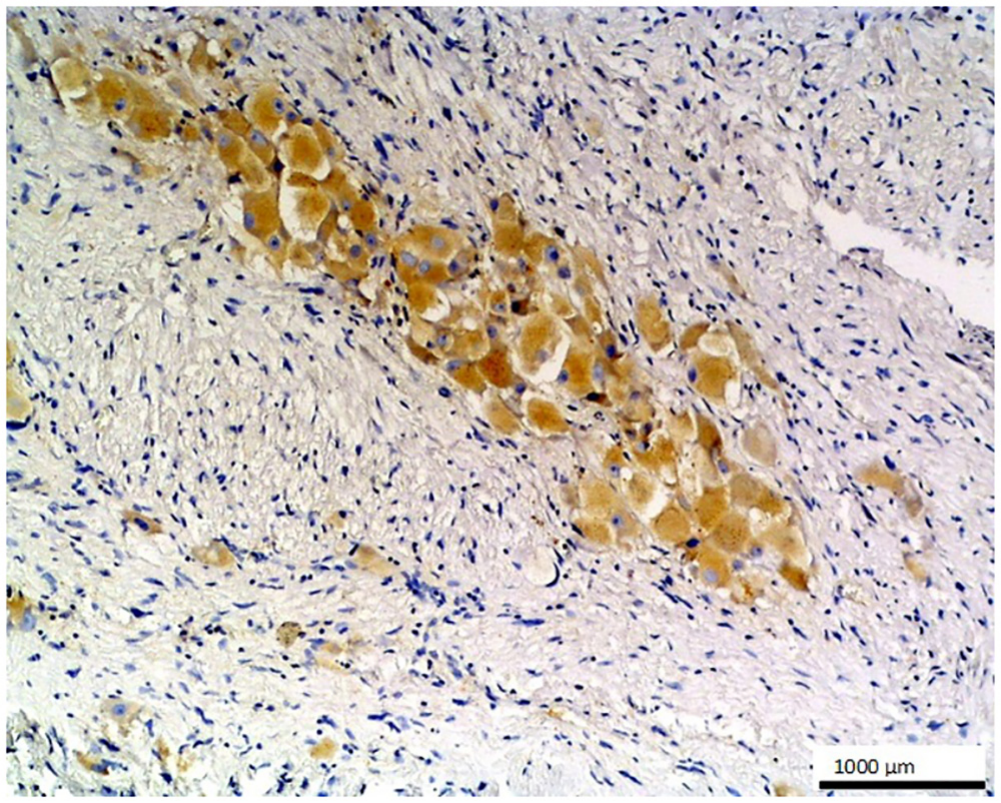
Ganglioneuroma. A positive moderate reaction against O56 antibody is seen in the ganglion cells of this benign tumor in the centre of microphotograph; LSAB, hematoxylin counterstained, magn. 100x.

**Fig 7:**
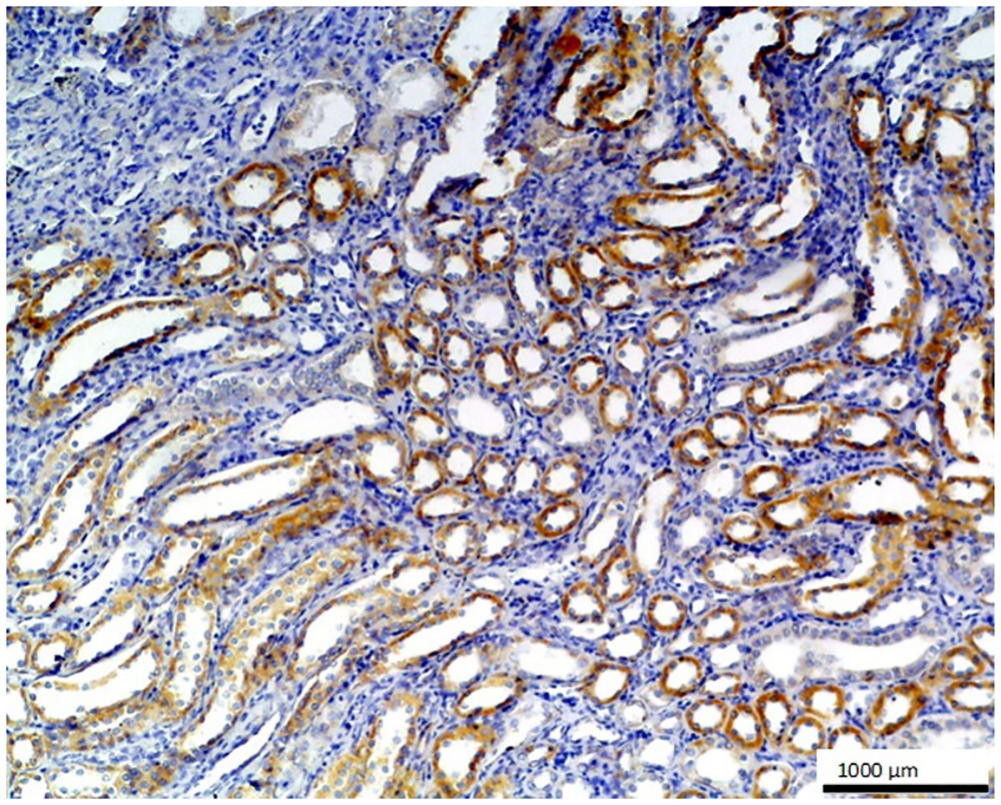
Renal graft chronic rejection. The cells of renal tubules show strong positive staining against O56 antibody; LSAB, hematoxylin counterstained, magn. 100x.

**Fig 8:**
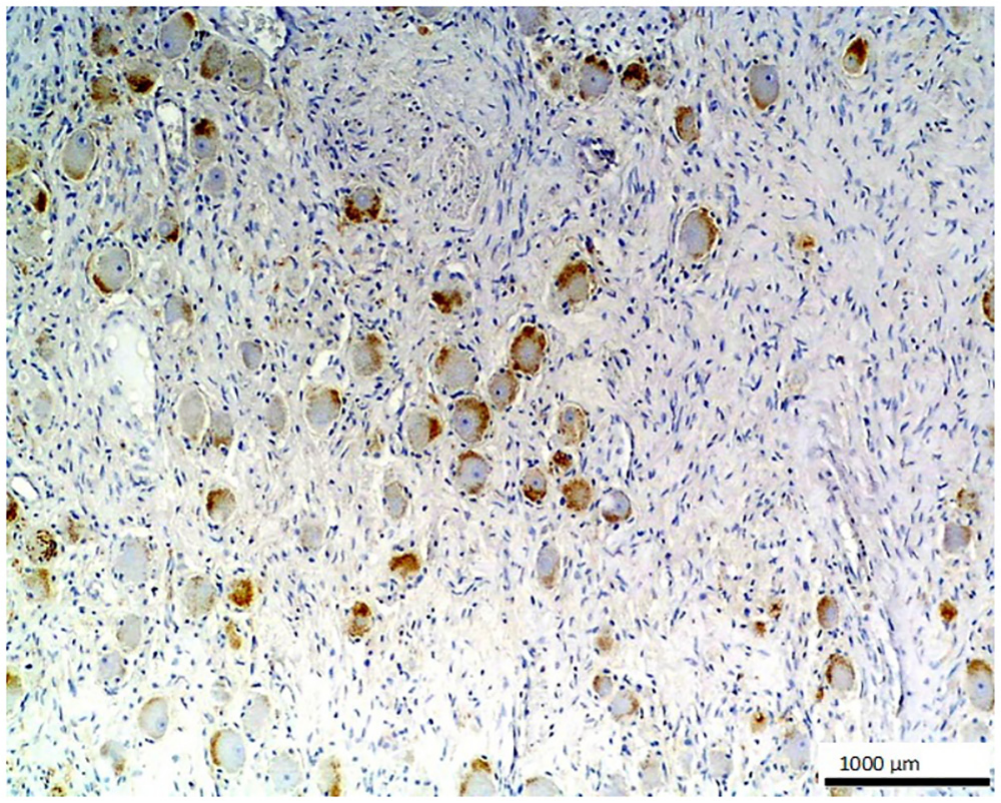
Neural ganglia. The cells of neural ganglia show a moderate positive reaction against O56 antibody; LSAB, hematoxylin counterstained, magn. 100x.

**Fig 9:**
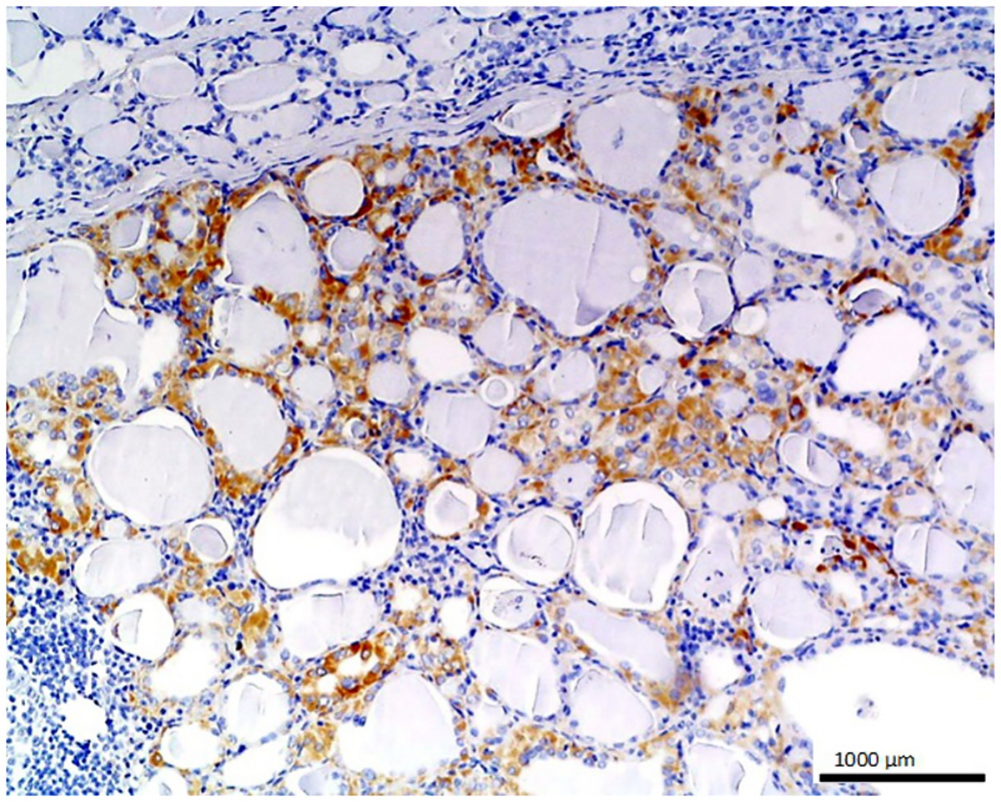
Thyroid gland. Thyrocytes of the normal thyroid gland show a strong reaction against O56 antibody; LSAB, hematoxylin counterstained, magn. 100x.

Both antibody preparations were then studied for their reactivity with antigens present in human tissue sections. For these experiments normal, healthy tissues were used as well as material obtained from rejected renal transplants, primary benign and malignant tumors and metastatic carcinoma. Results are shown in Figs 3–9.

**Fig 10:**
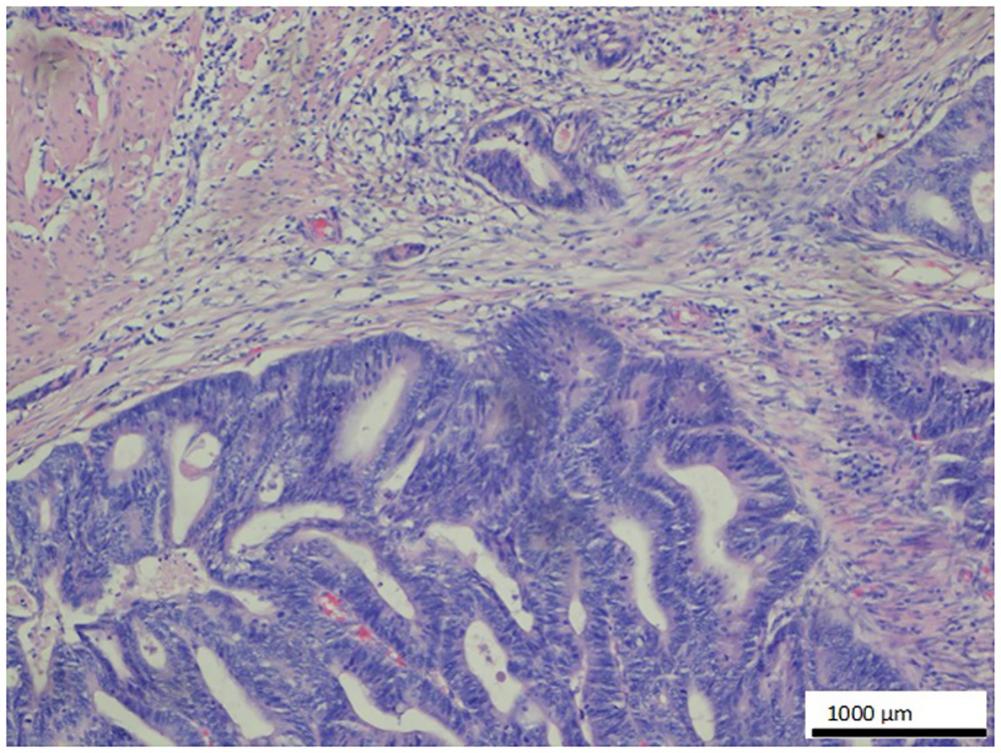
Negative control—no reaction with O56 antibody. Specimen from the colon adenocarcinoma G1 (counterstaining with hematoxylin-eosin), magn. 100x. First antibody O56 was omitted and replaced by TBS.

**Fig 11:**
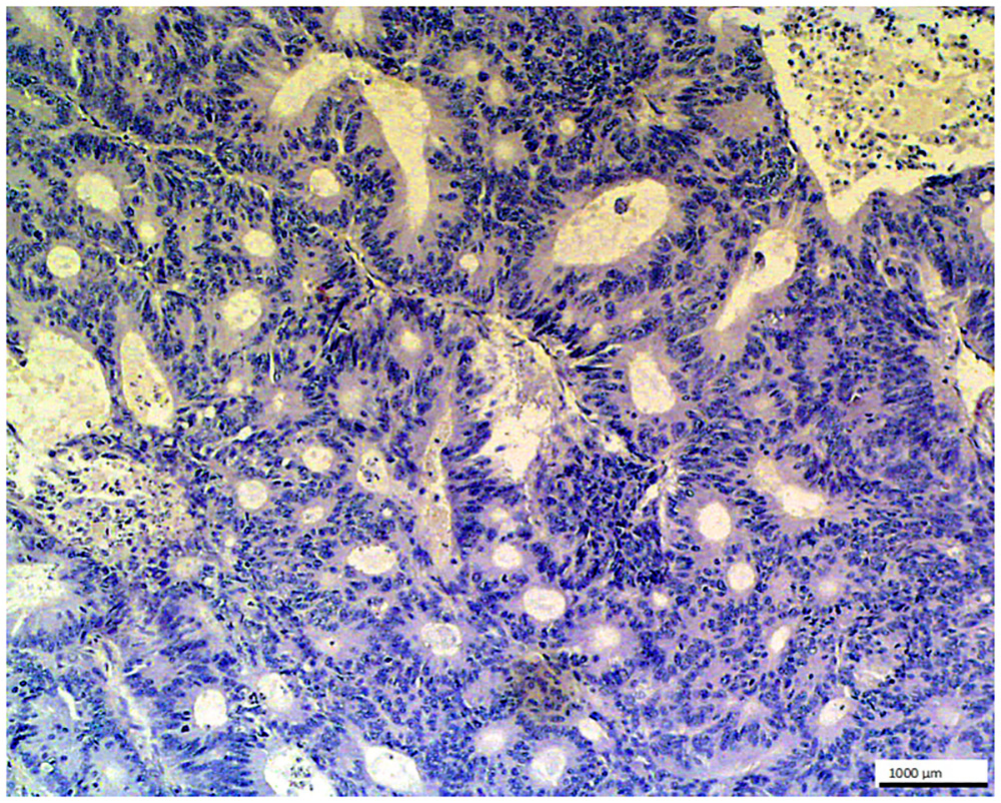
Metastatic colon adenocarcinoma in liver- control. No staining is seen in the glandular cells of this metastatic tumor with O56 antibody blocked with LPS O56; LSAB, hematoxylin counterstained, magnification 100x.

The negative control for the evaluation of the staining protocol was performed by omitting first anti-O56 antibodies (Fig 10) and by preblocking antibodies with LPS 056. As shown on Fig 11 labeling of metastatic colon adenocarcinoma in liver with anti-O56 antibodies was abolished when LPS O56 was added to the antibody sample.

No staining was observed using both antibodies on 1) normal tissues and cells: parathyroid gland, suprarenal gland, lymphatic or blood vessels, bronchial squamous epithelium, ovarian serous epithelium, bone, T and B cells, macrophages, granulocytes and nevoid cells, 2) benign tumors, e.g. in lipoma and papilloma, and 3) malignant tumors, such as gastrointestinal stromal tumor, nasopharyngeal carcinoma, lung microcellular carcinoma, basal cell carcinoma, liposarcoma [Table 1]. We noted an intense positive reaction with anti-*E*. *coli* O56 antibody in sections of liver metastasis from moderately differentiated colon adenocarcinoma where the glands are less regular (Fig 3). A similar reaction of this antibody was noted for pancreatic adenocarcinoma and slightly weaker for bronchoalveolar carcinoma and endometrial carcinoma (data not shown). These antibodies react with an as yet uncharacterized antigen present in hepatocytes as shown in Fig 4. Based on the granular intracytoplasmic pattern of immunostaining, we presume that the epitope may be localized in the cytoplasm [15]. Immunohistochemistry plays an important role in distinction of hepatocellular carcinoma (HCC) from other primary and metastatic neoplasms. Still no highly specific and sensitive marker for HCC has been identified. In this case an immunohistochemical collection, including multiple antibodies such as hepatocyte-specific antigen (Hepatocyte), pCEA, MOC-31, CD34, and TIF-1, should be used [16]. Based on this information, the antibody *E*. *coli* O56 could be useful in combination with other known markers rather than applied alone. Positive reaction of both anti-*E*. *coli* O56 and O24 antibodies has been revealed in a number of malignant tumors, e.g. hepatoblastoma, meningioma, and ganglioneuroblastoma, and a very strong positive reaction has been observed in cholangiocellular carcinoma cells (Fig 5). Interestingly, benign tumor, ganglioneuroma, from nervous tissue proves that tissue of neuroectodermal origin is recognized by anti-*E*. *coli* O56 antibody (Fig 6), while only trace reactivity with anti-*E*. *coli* O24 antibody could be recorded. The cells of normal renal tubules (Fig 7), ganglia cells (Fig 8) and thyrocytes of normal thyroid gland (Fig 9) were stained with both antibodies and expressed a much stronger reaction with *E*. *coli* O56 antibody. When considering that neuroectodermal tissues (ganglia, nerves and brain) develop from ectoderm, the reactivity of our antibodies in normal tissue (ganglia) as well as in tumor tissue (ganglioneuroma) is not surprising. Endodermal origin glands of the gastrointestinal tract, endocrine thyroid glands and organs have shown positive reactions with *E*. *coli* O56 antibody too. A positive reaction was also observed in myoepithelial cells of mixed tumor of the salivary gland. An interesting observation is that normal thymus, spleen, salivary gland and tonsil did not show immunoreactivity, which proves the consistency of our results.

**Table 1:** Reactivity of anti-O56 and anti-O24 antibodies with human tissues and cells.

	Human tissues and cells	Reactivity with anti-O56 antibodies	Reactivity with anti-O24 antibodies
Normal tissues and cells	Parathyroid gland	**-**	**-**
	Suprarenal gland	**-**	**-**
	Lymphatic vessels	**-**	**-**
	Blood vessels	**-**	**-**
	Bronchial squamous epithelium	**-**	**-**
	Ovarian serous epithelium	**-**	**-**
	Bone	**-**	**-**
	T cells	**-**	**-**
	B cells	**-**	**-**
	Macrophages	**-**	**-**
	Granulocytes	**-**	**-**
	Nevoid cells	**-**	**-**
	Thymus	**-**	**-**
	Spleen	**-**	**-**
	Salivary gland epithelial cells	**-**	**-**
	Tonsil	**-**	**-**
	Renal tubuli	**+**	**+**
	Ganglia cells	**+**	**+**
	Thyrocytes	**+**	**+**
	Thyroid gland	**+**	**-**
Benign tumors	Lipoma	**-**	**-**
	Papilloma	**-**	**-**
	Ganglioneuroma	**+**	**+/-**
Malignant tumors	Gastrointestinal stromal tumor	**-**	**-**
	Nasopharyngeal carcinoma	**-**	**-**
	Lung small cell carcinoma	**-**	**-**
	Basal cell carcinoma	**-**	**-**
	Liposarcoma	**-**	**-**
	Hepatoblastoma	**+**	**+**
	Meningioma	**+**	**+**
	Ganglioneuroblastoma	**+**	**+**
	Cholangiocellular carcinoma	**+**	**+**
	Metastatic colon adenocarcinoma in liver	**+**	**-**
	Pancreatic adenocarcinoma	**+**	**-**
	Bronchioloalveolar carcinoma	**+**	**-**
	Endometrial carcinoma	**+**	**-**
	Hepatocellular carcinoma	**+**	**-**

In general, *E*. *coli* O56 antibody performs better than *E*. *coli* O24 in staining the nervous tissues and epithelial tissues. Both nervous tissues, normal (ganglia) and tumor (ganglioneuroma), react with the antibody. Regarding epithelium, each type of this tissue has a different biological function and many of the biomarkers target specific proteins. Functional markers usually more frequently expressed by epithelial tumors are useful for immunohistochemical differentiation of metastatic tumors of unknown origin. The results of our experiments indicate that the glandular tumor, its metastasis, and also epithelium of renal tubules and glandular epithelium of the thyroid gland, were stained, but stratified epithelium of skin (results not shown) was not stained definitely. Based on these findings, we believe that *E*. *coli* O56 antibody could be considered as a promising biomarker for glandular epithelium after performing studies with a broader spectrum of clinical specimens. Therefore, the most relevant observation is that the epitope recognized by anti-O56 antibodies is a potential marker specific for glandular epithelium and nervous tissue.

**Fig 12:**
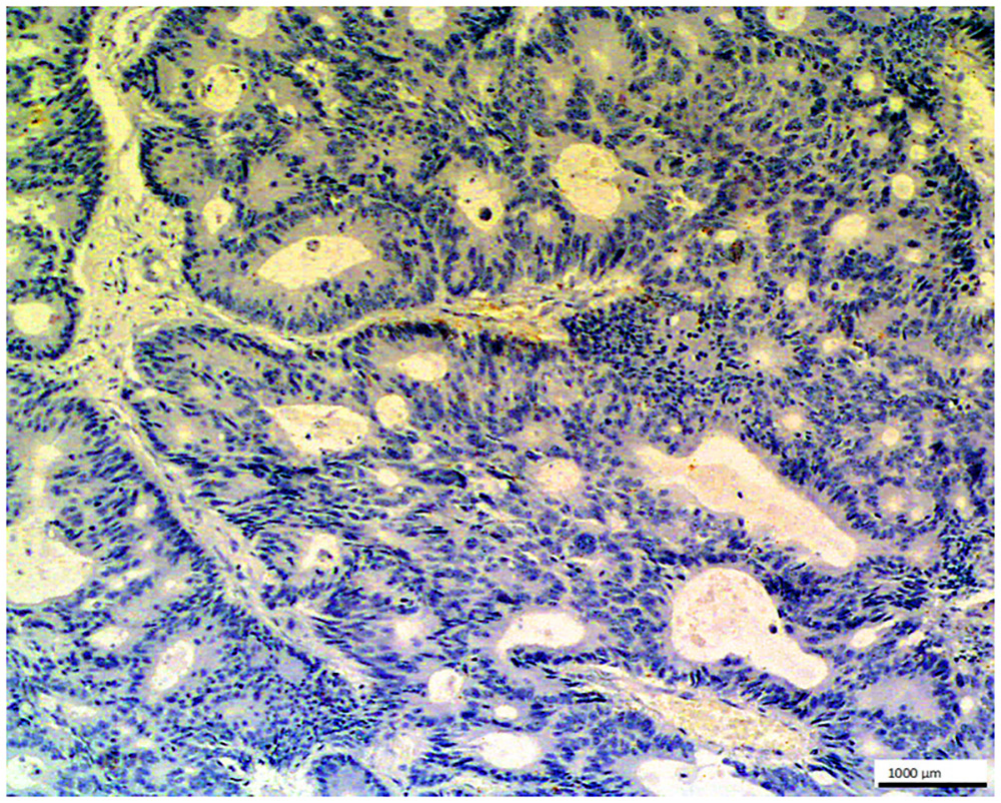
Metastatic colon adenocarcinoma in liver- control. No staining is seen in the glandular cells of this metastatic tumor with O56 antibody after periodate oxidation of tissue sample; LSAB, hematoxylin counterstained, magnification 100x.

To establish a character of the cross-reactive epitope the tissue sample with metastatic colon adenocarcinoma in liver (Fig 3) was subjected to the periodate oxidation procedure (2% sodium metaperiodate) before adding anti-O56 antibodies. The lack of stanining (Fig 12) strongly speaks for carbohydrate character of the cross-reactive epitope.

Further studies should be performed to determine the structure of the tissue epitope recognized by anti-*E*. *coli* O56 antibodies. In *E*. *coli* O56 LPS the sialic acid residue is glycosylated at position 7 [3]. Thus it will be important to establish whether structural homology occurs with tissue antigens or it concerns conformational type of mimicry. It is worth to notice that reactivity of both sera, anti-O56 and anti-O24, could be inhibited by a corresponding dimer of repeating O-specific units containing a glycosylated sialic acid residue [unpublished data]. This observation supports the conclusion that the cross-reactivity is associated with the presence of sialic acid that is a component of both: bacterial polysaccharides and human glycans. Further study will be conducted to elucidate a molecular basis of the described phenomenon. Interestingly, our results indicate that cross-reactive antibodies can be not only important for exploration of molecular mimicry and its implications for host-pathogen interactions but also may be an interesting subject of further studies oriented on their application in diagnostics.
